# Intrinsic Decomposition Method Combining Deep Convolutional Neural Network and Probability Graph Model

**DOI:** 10.1155/2022/4463918

**Published:** 2022-02-10

**Authors:** Yuanhui Yu

**Affiliations:** School of Computer Engineering, JiMei University, Xiamen 361021, Fujian, China

## Abstract

With the rapid development of computer vision and artificial intelligence, people are increasingly demanding image decomposition. Many of the current methods do not decompose images well. In order to find the decomposition method with high accuracy and accurate recognition rate, this study combines convolutional neural network and probability map model, and proposes a single-image intrinsic image decomposition method that is on both standard dataset images and natural images. Compared with the existing single-image automatic decomposition algorithm, the visual effect comparable to the user interaction decomposition algorithm is obtained, and the method of this study also obtains the lowest error rate in the quantitative comparison on the standard dataset image. The multi-image collaborative intrinsic image decomposition method proposed in this study obtains the decomposition result of consistent foreground reflectivity on multiple sets of image pairs. In this study, the eigenimage decomposition is applied to the illumination uniformity in the small change detection, and the promising reflectivity layer image obtained by the decomposition helps to improve the accuracy of the cooperative saliency detection. This study proposes an algorithm for the cooperation between CNN and probability graph model, and introduces how to combine the probability graph model with the traditional CNN to accomplish the pixel-level eigendecomposition task. This study also designs a single-image and multi-image intrinsic image decomposition results analysis experiments, then analyzes the probabilistic graphical model coordination intrinsic image decomposition results, and finally analyzes the convolutional neural network coordination intrinsic decomposition performance to draw the conclusion of this study. The effect on the Msrc-v2 dataset was increased by 0.8% over the probability plot model.

## 1. Introduction

Research on convolutional neural networks began in the 1980s and 1990s, and time delay networks and LeNet-5 were the first convolutional neural networks. After the 21st century, with the introduction of deep learning theory and the improvement of numerical computing equipment, convolutional neural networks have developed rapidly and have been used in computer vision, natural language processing, and other fields.

The intrinsic image decomposition problem was proposed by Barrow and Tenenbaum in 1978. They believe that when analyzing a scene, a series of essential features can be used to describe the scene. These essential features include the reflectivity of the surface of the object in the scene, and the object's process of restoring these features from the input image, such as geometry, scene depth information, direction, and color of incident illumination, is called the intrinsic image decomposition problem. For humans, intrinsic image decomposition is like an instinct. If you look at the object from any angle, no matter what kind of lighting conditions the object is having, people can easily identify the original color of the object, the geometry of the object, the direction of the light source, and the color of the light source. At present, the image forming model commonly used in the intrinsic image decomposition method is *I* = *R* × *S*, where *R* represents the reflectance layer image of the object, reflecting the reflection ability of the surface of the object to the illumination, and *S* represents the illumination layer image (shading). This model is the result of the interaction between the geometry of the object and the lighting, and × means multiplication by pixel.

In order to study the advantages and characteristics of the intrinsic decomposition method, many research teams at home and abroad began to conduct in-depth research on the decomposition method of this syndrome. In reference [1], the author proposes a fault type identification method based on symbol dynamic filtering (SDF) for early fault detection and intrinsic feature scale decomposition (ICD). The SDF is applied to extract fault features to describe bearing performance degradation, use cumulative and trigger fault alarms, decompose the extracted anomaly signals by ICD methods, and use the kurtosis method to select the main product components that contain most of the fault information for fault detection. The experimental results verify the effectiveness of the method in early detection and fault diagnosis of bearing faults. In reference [2], the author considers the decomposition of multi-component chirp signals (MCCSs) and develops a general model to characterize MCCSs, where the instantaneous eigen component (ICC) instantaneous frequency (IF) and instantaneous amplitude (IA) are modeled as a Fourier series. Therefore, the decomposition problem comes down to identifying the model that has been developed. The IF estimate is solved using a framework of general parametric time-frequency transforms, which can then be easily reconstructed by solving a linear system. In reference [3], the authors propose a spectral inheritance image decomposition (SIID) model that is designed to resolve natural scenes into purely independent internal components. The authors propose an efficient algorithm to decompose spectral images into their independent intrinsic components. To facilitate future SIID research, the authors also present a common data set with ground-based live illumination, shadows, reflections, and specular reflections, as well as meaningful error metrics, so that quantitative comparisons can be achieved. In reference [4], the author introduces the intrinsic image decomposition prior to the decomposition model for contrast enhancement. The author also regularizes the reflection layer into piecewise constants by introducing weighted l1 norm constraints on adjacent pixels based on color similarity, so that the resolved reflectivity is not greatly affected by the illumination information. The illumination layer is normalized by segmentation smoothing constraints. The proposed model is effectively solved by the Split Bregman algorithm. In reference [5], the authors design of a time-frequency analysis tool is still an open question, the tool can characterize the amplitude, frequency, and trend information of nonstationary plant-wide oscillations. The author proposes a new algorithm—multivariate intrinsic time scale decomposition (MITD). The screening process is added to the standard intrinsic time scale decomposition (ITD) to ensure that each decomposed product is a single component. Then, by solving the overdetermined linear equations, the MITD is extended from the modified ITD. In reference [6], the author proposes a new method, local mean decomposition (LMD). The LMD method can decompose seismic data into multiple product functions (PFs). Compared with the inherent mode functions (IMFs) of the EMD method, the PFs retain more details and the mode blending effect is weaker. The application of model data and field data shows that the LMD method can make the decomposition more accurate and capture the local features of seismic data at different time points. In reference [7], the authors found that variational mode decomposition (VMD) is a recently introduced adaptive data analysis method, which has attracted much attention in various fields. However, VMD is based on the assumption of the narrowband characteristics of the signal model. To analyze the wideband nonlinear chirp signal (NCS), the authors propose an alternative method called variational nonlinear chirped mode decomposition (VNCMD). In reference [8], the authors found that the machine-based single-image intrinsic decomposition (SIID) method decomposes the captured scene into its albedo and shadow images by using a large amount of known and realistic knowledge of ground truth decomposition. Collecting and annotating such data sets are two ways that cannot be extended to be sufficiently diverse and authentic, with two images that observe the same scene but with different illuminations providing useful information about their intrinsic properties.

Since deep learning methods can be used to learn rich feature representations in images, more and more scholars have applied the deep convolutional neural network (CNN) to the problem of monocular image depth estimation in recent years, which makes the research of this topic develop rapidly. The development of a more powerful representation of the map becomes an inevitable requirement for the development of the graph model, and the generation of the probabilistic graph model becomes the inevitable result of the development of the graph model. The intrinsic decomposition method combining deep convolutional neural network and probabilistic graphical model studied in this study is very effective for image intrinsic decomposition.

In order to recover the intrinsic image in the intrinsic image decomposition, the deep learning method and the probability map model have been widely studied in order to recover the intrinsic image. In reference [9], the author applies deep learning in the field of bioinformatics, through the field of bioinformatics (i.e., omics, biomedical imaging, and biomedical signal processing) and deep learning architecture (i.e., deep neural networks and convolutional neural networks), recursive neural networks, and emergency architecture to classify research. In addition, the author also discusses the theoretical and practical issues of deep learning in bioinformatics and proposes future research directions. In reference [10], the authors applied deep learning in cell imaging to discuss the application of this new analytical method in regulatory genomics and cell imaging. The authors provide a background in deep learning and can be successfully applied to obtain biological insights. In addition to providing specific applications and providing practical skills, the article highlights possible deficiencies and limitations to guide computing biologists when and how to take advantage of this new technology. In reference [11], the author applies deep learning to face recognition and proposes a hybrid convolutional network (ConvNet)-restricted Boltzmann machine (RBM) model for face verification. In order to characterize facial similarity from different aspects, the authors connect features extracted from different facial regions by different depth ConvNets. After pretraining each ConvNet and RBM separately, the entire hybrid network is jointly optimized to further improve accuracy. In reference [12], the author applies the probability map model to the neural network model and proposes a new recurrent neural network model based on the combination of echo state network (ESN) and dynamic Bayesian network (DBN). The author proposes a new graph-based echo state network (GESN) model for nonlinear system modeling. Finally, the performance of GESN is tested using Mackey–Glass time series and laser time series data prediction. Simulation and comparison results show that the model has a good application prospect. In reference [13], the authors used the exponential random graph model (ERGM) to explore the association between mental health and network structure and the association between adverse mental health and social isolation, using the Strengths and Difficulties Questionnaire (SDQ) to assess the school network, Social Demographic Association and Mental Health. The results show a homosexual pattern of sexual and mental health. In addition, participants with higher SDQ scores have a lower probability of a draw. In reference [14], the author applies the probability map model to algorithm research and proposes a damage-aware multicast routing algorithm based on the hypergraph model. The author proposes a new multi-domain hypergraph model that considers the Kpath strategy and the method of establishing interdomain supertree based on hyperpath. The experimental results show that the proposed multicast routing algorithm is effective. By measuring the effects of K value, multicast size, wavelength number, and distribution, good average blocking performance can be obtained. Most of the literature cited above are about convolutional neural networks and probabilistic models, but they are not comprehensive enough in image intrinsic decomposition, and are not applied in combination with convolutional neural networks and probabilistic graph models. This is the focus of this study.

In order to find the decomposition method with high accuracy and accurate recognition rate, this study combines the convolutional neural network and the probability map model. This study first proposes a hierarchical decomposition structure based on the zero norm sparse representation to establish nonlocal pixels. In this study, the concept of multi-image collaborative intrinsic image decomposition is proposed, which is to perform joint eigenimage decomposition on multiple images with the same foreground, background, and illumination, and to resolve the same foreground reflectivity in multiple reflectivity layer images. The values are consistent, that is, have the same color and brightness. In this study, superpixel is used to represent the reflectivity layer image, and then based on the sparse representation of zero norm, the reflectance relationship between image pixels is constructed with a unified framework. This study also proposes an algorithm for the cooperation between CNN and probability graph model, and introduces how to combine the probability graph model with the traditional CNN to accomplish the pixel-level eigendecomposition task. This study also designs an experiment to analyze the internal image decomposition results of a single image and multiple images, and then analyzes the internal image decomposition results of the probabilistic graphical model coordination. The image intrinsic decomposition method is based on deep convolutional neural networks and probabilistic graphical models.

## 2. Method

### 2.1. Convolutional Neural Network (CNN) Principle

#### 2.1.1. Convolution Layer

The different convolution kernels are filtered without interference with each other, each of which is an independent filter that effectively extracts a particular type of feature from the input image or a feature map output from the previous layer. In order to add nonlinear components to a purely linear convolution operation, an activation function is usually added after the convolutional layer to enhance the expressive power of the model. The process is as follows:

(1) $$\begin{array}{c} h_{j}^{1}=f\left(\sum_{i\in M_{j}}W_{ij}^{l}*x_{i}^{l-1}+b_{j}^{l}\right), \end{array}$$

where *f* is the activation function, *x* is the input set, W is the convolution kernel, *b* is the offset, and the initial value is 0. The output of the l-1th layer is convolved with a specific convolution kernel to obtain all the feature maps of the first layer, so the number of feature maps available for each layer is equal to the number of convolution kernels.

#### 2.1.2. Pooling Layer

The pooling layer is used to reduce the dimensionality of the feature map output by the convolutional layer, which greatly reduces the parameter size of the network and improves the overfitting problem common in CNN. Even if the input image is panned, scaled, or rotationally deformed, the pooled layer enables the entire CNN to maintain as much as possible the level of the mapping between the signature map and the supervisory label signal. There are three types of pooling methods, namely, maximum pooling, averaging pooling, and random pooling. The maximum pooling, as the name implies, is to calculate the pixel value of the entire calculation area by calculating the maximum value of the pixels in the small square area in the feature map, which can effectively reduce the deviation of the mean value caused by the convolutional layer parameter error, so that more texture information in the image can be preserved.

#### 2.1.3. Activation Function

The sigmoid function: a threshold function of the S-type exponential function, which is characterized by differentiability, monotonicity, smoothness, convenience, and derivation, and is prone to gradient disappearance. Its expression is as follows:

(2) $$\begin{array}{c} \sigma \left(x\right)=\frac{1}{1+e^{-x}}. \end{array}$$

Tanh function: a modified version of the function, with zero as the center of symmetry, with strong network fault tolerance, delaying the saturation period than the function. Its formula is as follows:

(3) $$\begin{array}{c} \tanh\left(x\right)=\frac{e^{x}-e^{-x}}{e^{x}+e^{-x}}. \end{array}$$

ReLu function: in the monocular image depth estimation task, ReLU will cause the sparsity of the probability of assigning depth values to pixels, implicitly trimming the less likely depth prediction values, which may have a positive effect. Its expression is as follows:

(4) $$\begin{array}{c} ReU\left(x\right)=\max\left(0,x\right). \end{array}$$

#### 2.1.4. Softmax Regression

The softmax regression layer transforms the linear output into a multi-probability distribution for image multi-classification problems. The softmax *p*(*z*)=(*p*_1_(*z*),…, *p*_*m*_(*z*)) regression is defined as follows:

(5) $$\begin{array}{c} p_{i}\left(z\right)=\frac{e^{z_{i}}}{\sum_{j=1}^{m}e^{z_{j}}},\left(i=1,\ldots ,m\right). \end{array}$$

#### 2.1.5. Loss Layer

In the previous work of monocular image depth estimation, the commonly used loss functions include as follows: mean-square error (MSE), scale-invariant loss function, and berHu loss. The mean square error loss expression is as follows:

(6) $$\begin{array}{c} MSE=\frac{1}{n}\sum_{i}^{n}{\left(y_{i}^{*}-y_{i}\right)}^{2}, \end{array}$$

where *y*^*∗*^ represents the true value and *y* represents the network prediction value. The scale constant loss function expression is as follows:

(7) $$\begin{array}{c} L\left(y,y^{*}\right)=\frac{1}{n}\sum_{i}d_{i}^{2}-\frac{\lambda }{n^{2}}{\left(\sum_{i}d_{i}\right)}^{2}, \end{array}$$

where *d*_*i*_=log  *y*_*i*_ − log  *y*_*i*_^*∗*^. This loss, while using the Euclidean distance loss, incorporates the information retained by the deep learning network on the three-dimensional structure of the scene during prediction, which enhances the stability of CNN adaptation to different scenarios to some extent.

### 2.2. Probability Graph Model

#### 2.2.1. Undirected Graph Model

In the undirected graph, if the node xj is the neighboring node of xi, there is only one undirected edge between them. When the Markov property in the Markov random field gives the neighborhood of the random variable, the random variable xi and other random variables are conditionally independent.

(8) $$\begin{array}{c} P\left(X_{i}|\frac{X}{X_{i}}\right)=P\left(X_{i}|X_{N_{i}}\right), \end{array}$$

where *N*_*i*_ represents the neighborhood of *X*_*i*_ and *X*\*X*_*i*_ represents all nodes in *X* except *X*_*i*_. Based on the Hammersley–Clifford theorem, the joint probability distribution of Markov random field modeling can be seen as a Gibbs distribution, namely

(9) $$\begin{array}{c} P\left(X,Y\right)=\frac{1}{z}\exp\left(\frac{-\Sigma_{cec}v_{c}\left(x_{c},x_{e}\right)}{T}\right), \end{array}$$

where *c* is a group, it is a connected subgraph, *V*_*c*_(*X*_*c*_, *Y*_*c*_) is the energy function of the set of random variables defined in group *c*, and *Z* is a normalization factor, which can be marginalized in the Markov random field. All random variables are obtained. In the Markov random field, the size of the group has an important influence on the computational complexity. In practice, researchers often use low-order neighborhood systems. In the Pairwise Markov Random Fields model, the joint probability distribution is expressed as follows:

(10) $$\begin{array}{c} P\left(X,Y\right)=\frac{1}{z}\prod_{i}\emptyset \left(X_{i},Y_{i}\right)\prod_{<i,j{>}_{j}\in N_{i}}\varphi \left(X_{i},X_{j}\right), \end{array}$$

where Φ(*x*_*i*_) is a one-dimensional potential function, which is only related to the tag random variable *x*_*i*_. Similarly, Φ(*x*_*i*_, *x*_*j*_) is a binary potential function that reflects the relationship between the labels of *x*_*i*_ and *x*_*j*_.

#### 2.2.2. Directed Graph Model

One of the most used models in the directed graph model is the Bayesian network. The Bayesian network is a directed acyclic graph, and the directed edges in the graph illustrate the causal relationship between these nodes. The graph structure of the Bayesian network represents the conditional independence relationship between these nodes. Given the parent node of a node, this node condition is independent of its nondescendant node. Based on these conditional independent relationships, when giving the parent node of the node, the joint probability of all nodes can be factored into the product of the conditional probability of each node, that is, $P\left(\left\{X_{i}\right]\right)=\prod_{i}P\left(X_{i}\right)|pa\left(X_{i}\right)$, where pa(*X*_*i*_) represents the parent node of *X*_*i*_. This factorization simplifies parameter learning and reasoning in the Bayesian network model.

### 2.3. Single-Image Intrinsic Image Decomposition

#### 2.3.1. Decomposition Model

I represents the input image, and *S* and *R* represent the corresponding illumination layer and reflectance layer images, respectively. The intrinsic image decomposition model *I* = *S* × *R* expressed as *I* = *S* + *R* in the logarithmic domain (for the sake of simplicity, *I*, *S*, *R* are still used to represent the value of the logarithmic domain). The energy function of the intrinsic image decomposition of a single image is defined as:

(11) $$\begin{array}{c} F\left(S,R\right)=f_{s}\left(S\right)+f_{r}\left(R\right)+f_{a}\left(S\right). \end{array}$$

Among them, *f*_*s*_(*S*), *f*_*r*_(*R*), and *f*_*a*_(*S*) represent the light slowly varying constraints, global reflectivity constraints, and global scale constraints, respectively. Specifically, for the illumination layer, as with the Retinex algorithm, since the surface of the object generally changes smoothly and the illumination intensity of the incident light at each point is also the same, the illumination values of adjacent pixels are similar. *f*_*s*_(*S*) is defined as follows:

(12) $$\begin{array}{c} f_{s}\left(S\right)=\sum_{i↔j}w_{ij}^{S}{\left(S_{i}-S_{j}\right)}^{2}, \end{array}$$

where *i* ↔ *j* represents a pair of neighboring pixels *i* and *j*. *S*_*i*_ and *S*_*j*_ represent the illumination values of pixel *i* and pixel *j*. *w*_*ij*_^*S*^ measures the similarity of neighboring pixels *i* and *j*, which is defined as follows:

(13) $$\begin{array}{c} w_{ij}^{S}=e^{-{\left(Y_{i}-Y_{j}\right)}^{2}/\sigma_{i}^{2}}, \end{array}$$

where *Y*_*i*_ is the luminance value of pixel *i*, and *σ*_*i*_^2^ is the variance of the luminance values of all pixels in the window. We construct its illumination similarity matrix WS = *w*_*ij*_^*S*^ for input image I.

#### 2.3.2. Constructing a Reflectivity Layer Constraint Based on the Zero Norm Sparse Representation

Suppose image *I* contains *N* pixels, and each pixel is characterized by a reflectivity value (including *R*, *G*, and *B* channels) of all pixels in a window of size *K* centered on this pixel. The reflectance value of each pixel is initialized to its chrominance value. For pixel *i*, its chrominance value *C*_*i*_ is as follows:

(14) $$\begin{array}{c} C_{i}=\left[\frac{I_{i}^{r}}{I_{i}^{r}+I_{i}^{g}+I_{i}^{b}},\frac{I_{i}^{g}}{I_{i}^{r}+I_{i}^{g}+I_{i}^{b}},\frac{I_{i}^{b}}{I_{i}^{r}+I_{i}^{g}+I_{i}^{b}}\right]. \end{array}$$

Among them, *I*_*i*_^*r*^, *I*_*i*_^*g*^, and *I*_*i*_^*b*^ represent the *R*, *G*, and *B* values of pixel *i*, respectively. The chromaticity value represents the color value normalized by the illumination intensity, and some of the illumination changes can be removed to some extent. The mid chrominance value is used to approximate the reflectance value of the pixel. However, many colors such as white, black, and gray have little difference in their chromaticity values, so these colors cannot be distinguished by only the chromaticity features. While the real scene contains a large amount of white and black, it is possible to decompose the image of the real scene using only the eigenimage decomposition algorithm of the chroma feature.

#### 2.3.3. Model Solving

Using I and S to represent the reflectivity layer image *R*, that is, *R* = I − S, the specific expansion of each constraint in the energy function can be written as follows:

(15) $$\begin{array}{c} F\left(S\right)=\sum_{i\mapsto j}w_{ij}^{S}{\left(S_{i}-S_{j}\right)}^{2}+\sum_{i∼j}w_{ij}^{R}{\left(\Delta I_{ij}-S_{i}+S_{j}\right)}^{2}+\sum_{i\in ℬ}S_{i}^{2}, \end{array}$$

where ∆Ii*j* = Ii − Ij. Now F(S) is a quadratic function on the image S of the illumination layer. After derivation, it can be concluded that A has the following form:

(16) $$\begin{array}{c} A=4L\left(W^{S*}\right)+4L\left(W^{R*}\right)+2B. \end{array}$$

### 2.4. Multiple Image Collaborative Eigenimage Decomposition

#### 2.4.1. Image Model Based on Single-Color Ambient Light

It is assumed that the illumination layer image can be obtained by multiplying a global ambient light color component *l*_*e*_ by a local illumination magnitude *M* (shading magnitude), wherein for each picture, each pixel corresponds to its own *M*, but the *l*_*e*_ component is the same. *I* represents the input image, and *p* represents one pixel. The mathematical value of the illumination value *S*_*p*_ of the pixel *p* is then defined as follows:

(17) $$\begin{array}{c} s_{p}=l_{e}M_{p}. \end{array}$$

The ambient illumination *l*_*e*_ is a three-dimensional vector containing *R*, G, and B components, and *M*_*p*_ is a nonnegative scalar value representing the magnitude of illumination at *p*. Based on the assumption of a single color of light, the mathematical representation of the image forming model is *I*_*p*_ = *l*_*e*_*M*_*p*_*R*_*p*_, where *R*_*p*_ represents the reflectance value of pixel *p*. After inspection, it is determined that the revised content is consistent with the original intention of the author.

(18) $$\begin{array}{c} I_{p}=l_{e}+M_{p}+R_{p}. \end{array}$$

#### 2.4.2. Co-Retinex Cooperative Eigenimage Decomposition Model

The objective function of the collaborative eigenimage decomposition model is as follows:

(19) $$\begin{array}{c} E\left(l_{e},M,R\right)=E_{c}\left(l_{e},M,R\right)+\lambda_{m}E_{m}\left(M\right)+\lambda_{r}E_{r}\left(R\right)+\lambda_{e}E_{e}\left(M\right). \end{array}$$

*E*_*c*_ is the constraint of the image forming model, *E*_*m*_ is the constraint that constrains the slow variation of the illumination amplitude in one image, and *E*_*r*_ represents the correlation of the reflectivity of two superpixels within the same image or between images. The relationship between superpixels passes. The zero norm sparse representation is established. Finally, *E*_*e*_ constrains the global scale problem. *λ*_*m*_, *λ*_*r*_, and *λ*_*e*_ are both positive numbers, indicating the weight of each term. In the experiment, we set *λ*_*m*_ = 10, *λ*_*r*_ = 100, and *λ*_*e*_ = 1000.

For the two images in the pair of images, there are some constraints on the reflectivity or illumination between the two nonlocal pixels. It should be emphasized that the two nonlocal pixels mentioned herein may be two pixels adjacent or not adjacent to each other in the same image, or may be two pixels distributed in different images. In order to express simplicity, in the objective function, we only use one symbol, such as *l*_*e*_, to represent the variables corresponding to the two images, such as *l*_*1e*_ and *l*_*2e*_.

## 3. Experiment

### 3.1. Data Source

#### 3.1.1. UIUC Sports Data Set

The UIUC sports data set contains 8 categories: badminton, bocce, croquet, polo, rock climbing, rowing, sailing, and snowboarding (snowboarding). There are 800 images of each type, a total of 6400 images, and 200 samples of each class are randomly selected for testing, and the rest are training.

#### 3.1.2. Msrc-v2 Data Set

The Msrc-v2 data set is currently one of the more well-tested data sets for semantic segmentation and classification. The original database consists of 591 images, and the scene categories and semantic annotation statistics are listed in Table 1. For a better comparison, the number of training and test sets is 335 for training data and 256 for test data. Single images have a labeling category from 1 to 7, for a total of 22 (including background).

### 3.2. Evaluation Criteria

For the real vector *X* and the vector ^*X* obtained by the algorithm, the local mean square error calculation formula is as follows:

(20) $$\begin{array}{c} MSE\left(x,\hat{x}\right)={\left\|x-\hat{\alpha }\hat{x}\right\|}^{2}. \end{array}$$

Among them, $\hat{\alpha }=\arg\min_{\alpha }{\left\|x-\alpha \hat{x}\right\|}^{2}$. Given the true luminance image *S* and the luminance image $\hat{S}$ to be evaluated, the author defines the local mean square error as the sum of the LMSEs of all local windows of size *k* × *k* in the image, where the step size is *k*/2, the formula is as follows:

(21) $$\begin{array}{c} LMSE_{k}\left(S,\hat{S}\right)=\sum_{w\in W}MSE\left(S_{w},{\hat{S}}_{w}\right). \end{array}$$

The final score of the evaluation eigenmap algorithm is obtained by obtaining the LMSE from the albedo eigenmap and the luminance eigenmap according to the above formula, taking the average of the two as the final score. At the same time, it is normalized to get the maximum score for the evaluation score of 0 for the eigengram.

## 4. Results and Discussion

### 4.1. Analysis of Single-Image Intrinsic Image Decomposition Results

In Table 2, we present an example for each type of image in the Msrc-v2 data set. Here, GT represents the standard true value of *R* and *S* for the given image of the data set, and CR and CFS represent the Color Retinex method. From the results, we can see that in the examples of “panther” and “turtle,” both CFS and our method can effectively separate the reflectivity layer of the object from the illumination layer, while CR does not. In the “cup1” example, the light layer image obtained by our method has no reflectivity information, which is very close to the standard real *S*. Obviously, our approach surpasses other methods in both decomposition accuracy and visual comparison.

Table 3 gives the LMSE quantitative comparison results of the single-image intrinsic image decomposition method on the Msrc-v2 data set with other methods. In the 16 images contained in the Msrc-v2 dataset, our method yielded the lowest LMSE value in 10 images. The LMSE average for this method is 0.021, which is lower than 0.030 for the CR method and 0.025 for the CFS method. In addition, our approach yields very high performance on some examples where chroma features are not very efficient, such as “turtle,” “frog 2,” and “teabag 1.” This shows that our method relies on chromaticity features at a lower level, which will contribute to the decomposition of the intrinsic image of natural images in real scenes.

In addition to the Msrc-v2 dataset, we also compare the two existing intrinsic image decomposition methods that require user interaction on natural images. These two methods use three user interactions, that is, the user marks the reflectivity. The same pixel, the same pixel, and the brightest pixel are used as constraints to solve the intrinsic image decomposition problem. Our method can decompose the natural image eigenstate to obtain a globally consistent illumination layer and reflectivity layer image, and as an automatic method can obtain comparable results with the user interaction method.

### 4.2. Analysis of Multiple Image Collaborative Intrinsic Image Decomposition Results

The two images in a pair of images have this apparent illumination change. We compare with the existing single-image intrinsic image decomposition methods, namely CR and CFS. Both of these methods work independently on the two images in the image pair.

**Figure 1:**
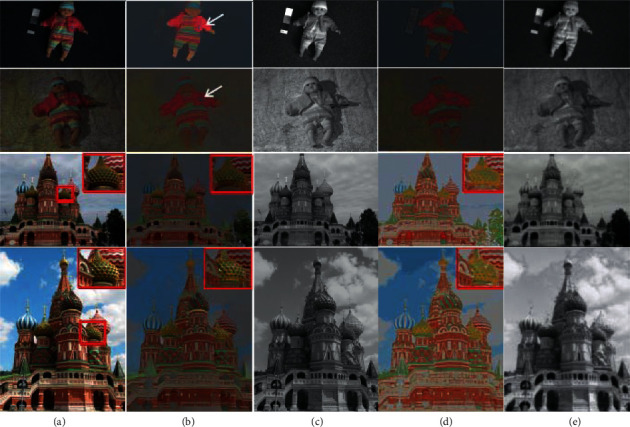
Compares the decomposition results of the eigenimage decomposition method with the single-image intrinsic image decomposition method CR on the image pair. (a) Contains input image pairs “doll” and “St Basile.” (b,c) The reflectance layer image and the illumination layer image obtained by CR. (d,e) The reflectance layer image and the illumination layer image obtained by the decomposition of the intrinsic image. Compared with CR, the same foreground in the reflectance layer image obtained by the method in this study is consistent in brightness and color.

The results of the comparison are shown in Figure 1. It is obvious that the intrinsic image decomposition algorithm of a single image cannot make the same foreground consistent on the reflectivity layer image corresponding to the two images of the image pair. For example, in CR, in the first pair of images “doll” in Figure 1, the same foreground “doll” on the reflectivity layer in the first image is significantly more than the “doll” luminance value on the reflectance layer image in the second image. Also, the area corresponding to the red frame in Figure 1 is inconsistent in the reflectance layer image. Even within the same image, these two methods do not result in consistent reflectivity for the two parts of the object that are far apart. For CFS, the effect of illumination or shadow remains on the reflectance image because it does not directly constrain two pixels that are not adjacent. Although we constructed the constraint on the reflectivity between two nonadjacent pixels, only the textured pixels are considered. Therefore, the effect on the single-color object is not good, and the image still has the effect of illumination. Our method uses chrominance features to directly construct the association between nonlocal pixels. As our results show, if the images are far apart or if the two pixels between the images have similar chromatic values, then our approach allows them to maintain consistent reflectivity.

Next, we quantify the results of the decomposition-integrated eigenimage decomposition by calculating the similarity of the same objects in the decomposition layer obtained by the decomposition. For each image on the image pair, we can get the foreground from the existing mask. After getting the foreground, we construct a color histogram to represent the foreground, and then use the cosine value to measure the similarity between the two foregrounds. The higher is the similarity, the more consistent is the reflectivity of the foreground representing the same decomposed object. Specifically, we quantize each channel of RGB onto the *M* segment, and the dimension of the entire color histogram is M3.  Table 4 lists the similarities of the same foreground in the reflectance layer images produced by the different methods. It can be seen that the method of this study achieves the highest similarity across all test images.

### 4.3. Analysis of Probabilistic Graph Model Coordinated Intrinsic Image Decomposition Results

**Figure 2:**
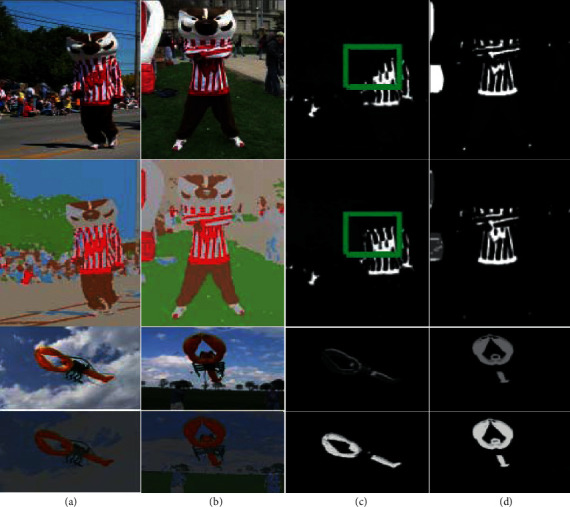
Comparison of synergistic significance detection results on the original image and the reflectivity layer image. The figure contains two pairs of images “Bucky” and “Kite.” For each pair of images, the first line includes the input image pair and the synergistic significance detection results obtained on the input image pair. The second row includes the reflectivity layer image obtained by the decomposition of the intrinsic image and the synergistic significance detection result obtained on the reflectance layer image.

A comparison of the results of the synergistic significance detection on the reflectance layer image pairs obtained from the input image versus the “Bucky” and “Kite” original images and the synergistic intrinsic image decomposition herein is shown in Figure 2. The collaborative saliency method used in this study is medium; Fu et al. establish a collaborative saliency detection model from the three characteristics of contrast, spatial position, and correspondence between images as shown in Figure 2.

**Figure 3:**
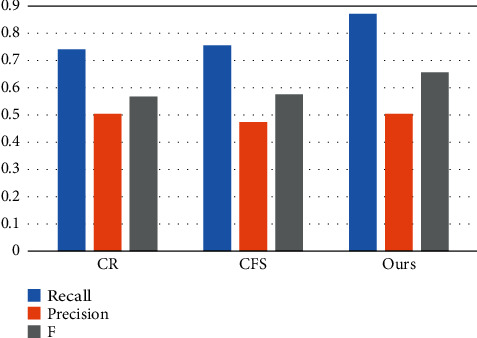
Collaborative algorithm comparison analysis chart.

It can be observed from the first line in Figure 2(a) that when the synergistic saliency detection is performed on the original image pair, since the left shoulder portion of the first figure character in the image pair has a shadow, the shadow occlusion in the synergistic saliency result part of it is not detected (the part of the green frame in the picture). On the second line, the reflection effect layer image pair with the illumination effect removed, the shadow effect is removed, so the left shoulder portion of the task in the first image is also detected in the synergistic significance result of the reflectance layer image (green frame in the figure part). For the original image of the first row of Figure 2(b), since the brightness of the foreground in the two images is very different, the detected significance values differ greatly on the synergistic significance result map on the original image. On the second line of the reflectance layer image, the synergistic significance results are consistent. In addition, Figure 3 is the accuracy of the results of the synergistic significance test of the original image pair and the reflectivity layer image pair of all test images. Compared with the F1 metric, it can be seen that the prospects obtained by the collaborative eigenimage decomposition are consistent. The reflectance layer image can improve the synergistic significance detection result.

### 4.4. Convolutional Neural Network (CNN) Coordinated Intrinsic Decomposition Performance Analysis

**Figure 4:**
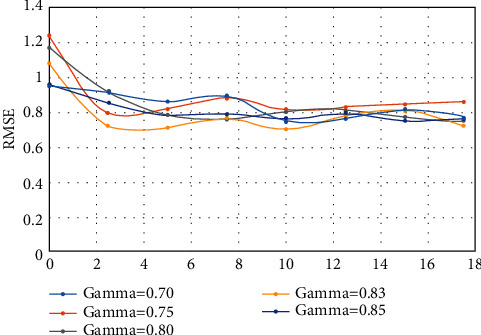
RMSE plot of CNN model output based on different thresholds *γ*.

Figure 4 shows the RMSE plot of the output of the NSW-CNN model for different gamma values at *m* = 120. It can be seen from the figure that when the *γ* value is less than or equal to 0.8, since the threshold is too small, the model considers that many superpixel blocks not belonging to the same scene are considered to belong to the same scene and performs image smoothing filtering, which deviates from the correct scene depth value and predicts depth. The RMSE of the graph fluctuates significantly, and the quality of the depth map decreases. When the threshold exceeds 0.83 and is set to 0.85, the algorithm determines whether the similarity requirements are too strict, and many superpixel blocks belonging to the same scene are considered not to belong to the same scene, and the predicted depth map quality is lowered again. It can be seen that the algorithm can fully utilize the depth information of adjacent superpixel blocks to enhance the smoothness of the depth map of the entire scene when the threshold is *γ* = 0.83.

Table 5 lists the magnitudes of performance indicators such as RMSE and REL from the image depth estimation model when *γ* takes different values. Obviously, with the addition of the NSW-CNN module, almost all of the performance of the predicted depth maps has improved, especially with RMSE down 8% and REL down 7%. It intuitively reflects that the NSW algorithm uses the similarity of adjacent superpixel blocks to effectively enhance the sharpness of the edge of the contour of the scene, and the accuracy of the relative depth also increases, closer to the actual depth map label.

## 5. Conclusions

In order to find an intrinsic decomposition method that combines convolutional neural network and probability map model to improve the accuracy and recognition efficiency of eigendecomposition, this study draws the following conclusions:

1. This study proposes a single-image eigenimage decomposition method based on a hierarchical structure. The hierarchical structure not only improves the efficiency of decomposition, but also makes the algorithm not rely too much on chrominance features. Based on the constraints on the reflectivity layer and the illumination layer, the single-image intrinsic image decomposition method proposed in this study achieves better than the existing single-image auto-decomposition algorithm on both the standard dataset image and the natural image. The user interaction decomposition algorithm can compare the visual effects, and in the quantitative comparison on the standard dataset image, the method of this study also obtains the lowest error rate.
2. In this study, the intrinsic image decomposition algorithm is extended to multiple images with the same foreground, illumination, and background, and the concept of collaborative intrinsic image decomposition is proposed. This study proposes two ways to reduce the constrainedness of the cooperative intrinsic image decomposition constraint based on superpixel to represent the reflectivity layer image and the reflectivity layer image constraint established by sparse representation.
3. The multi-image collaborative intrinsic image decomposition method proposed in this study obtains the decomposition result of the same foreground reflectivity on multiple sets of image pairs. In this study, the eigenimage decomposition is applied to the illumination uniformity in the small change detection, and the promising reflectivity layer image obtained by the decomposition helps to improve the accuracy of the cooperative saliency detection.
4. An algorithm for coordinating CNN and probability graph models is proposed. In order to make full use of the closely related feature information of adjacent pixels to improve the accuracy of the predicted depth map, it is introduced how to combine the probability map model with the traditional CNN to accomplish the pixel-level intrinsic decomposition task. In the custom CNN layer, the similarity is used as the filter function content to filter the rough estimate depth map outputted by the probability map model, and the smoothness of the depth map prediction of the depth map is enhanced. Then, the probability map model network is input from the low resolution. In the depth map, the depth map of the scene edge is restored, and the depth map of the scene is optimized twice. The effect on the Msrc-v2 dataset was increased by 0.8% over the probability plot model.

## Figures and Tables

**Table 1:** Msrc-v2 dataset scenario, labeling, and segmentation category statistics.

Semantic segmentation and labeling categories	Scene recognition
Building, grass, tree, cow, sheep, sky, airplane, water, face, car, bicycle, flower, sign, bird, book, chair, road, cat, dog, body, boat, background	Sign, bird, dog, cat, bicycle, tree, water, sheep, person, building, cow, chair, airplane, grass, city, flower, book, boat, nature, car, face

**Table 2:** Comparison of intrinsic image decomposition results of three methods in different images in the Msrc-v2 data set.

Images	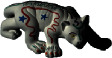	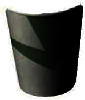	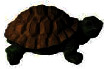

GT	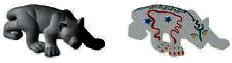	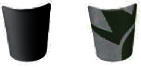	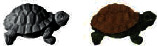

CR	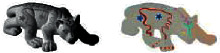	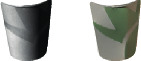	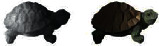

CFS	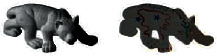	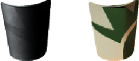	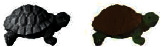

Ours	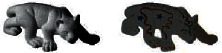	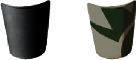	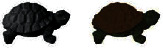

**Table 3:** Quantitative comparison of intrinsic image decomposition results on all images in the Msrc-v2 data set by different methods.

	Box	Cup 2	Deer	Dinosaur	Frog 1	Frog 2	Paper 1
CR	0.013	0.011	0.041	0.035	0.066	0.071	0.004
CFS	0.005	0.005	0.045	0.026	0.051	0.069	0.008
Ours	0.007	0.004	0.042	0.028	0.050	0.046	0.002
	Paper 2	Raccoon	Squirrel	Sun	Teabag 1	Teabag 2	
CR	0.004	0.015	0.072	0.003	0.041	0.023	
CFS	0.005	0.004	0.074	0.002	0.042	0.017	
Ours	0.005	0.004	0.073	0.003	0.020	0.026	

**Table 4:** Comparison of foreground similarity between two reflectivity layer images obtained by different methods.

	CR	CFS	Ours
Kite	0.00073	0.0009	0.3043
Doll	0.00017	0.001968	0.05177
St Basile	0.01347	0.005790	0.03337
Bucky	0.01122	0.01522	0.03367

**Table 5:** Comparison of intrinsic decomposition performance before and after using CNN model.

	Error (the lower the better)			Accuracy (the higher the better)		
	RMSE	REL	Log10	*δ* < 1.25	*δ* < 1.25 × 2	*δ* < 1.253 × 3
Intrinsic decomposition	0.692	0.310	0.074	0.652	0.873	0.904
Ours (+CNN)	0.684	0.303	0.075	0.663	0.871	0.912

## Data Availability

The data that support the findings of this study are available from the corresponding author upon reasonable request.
